# The Goldilocks Dilemma in Acute Ischemic Stroke

**DOI:** 10.3389/fneur.2013.00164

**Published:** 2013-10-21

**Authors:** Aaron P. Tansy, David S. Liebeskind

**Affiliations:** ^1^Department of Neurology, UCLA Stroke Center, University of California, Los Angeles, CA, USA

**Keywords:** stroke, therapy, endovascular, perfusion, ischemia

## Abstract

Despite the advent of and exciting advances in novel endovascular therapies, t-PA remains the only proven treatment for acute ischemic stroke to date. Although a variety of reasons likely underlie why past trials of endovascular strategies have been unsuccessful, we address in this perspective piece one critical unknown for which a solution is undoubtedly necessary if future ones are to meet with success: determination and selection of patients that are “just right” for endovascular treatments, or the Goldilocks dilemma. Key clinical criteria highlighted in past trials may help provide a solution to this critical problem. However, for them to do so, we propose that they must be applied in service of a model that accounts for the nuanced, dynamic nature of acute ischemic stroke better than the prevailing “time is brain” model. We provide and examine three clinical cases to illustrate this proposal towards solving the Goldilocks dilemma and advancing treatment in acute ischemic stroke. Further, we address our field’s ongoing challenge and mission in the meantime to best care for the “not-so-right” patients, by far the majority of the affected stroke population.

The introduction of intravenous tissue plasminogen activator (t-PA) heralded a sea change in the management of acute ischemic stroke (AIS). For the first time ever, a medical therapy for acute stroke was proven effective in reducing long-term impairment (1). Since that landmark event nearly two decades ago, the AIS field has witnessed the exciting development of novel endovascular strategies and heightened hopes of potentially improving upon t-PA’s clinical efficacy (2–7). The Goldilocks dilemma, or finding the ideal patient who is “just right,” remains the most formidable challenge in establishing new therapies for AIS.

Unfortunately, as of yet, no endovascular method has demonstrated itself more clinically effective than t-PA either in head-to-head comparison or in combination with it (5–7). Why is this? This question is an undoubtedly challenging and complex one, but also an undoubtedly necessary one to answer. Indeed, continued progress in AIS treatment and any potential role that endovascular methods may play in it rests in the balance. We address the Goldilocks dilemma as a key unsolved piece of this larger problem that has already received a great deal of attention in the stroke community: identifying the ideal patients and enrolling them in clinical trials seeking to prove efficacy of endovascular treatments.

The recent disappointing outcomes in related trials are a reflection not necessarily of flawed endovascular therapies, but, rather, of flawed selection of candidates likely to benefit from them. We also propose that improvement of the theoretical framework of ischemic stroke on which the criteria for determination of “just right” trial-eligibility is based may allow future trials to finally achieve success. Finally, we emphasize that, although our Goldilocks search for the “just right” acute stroke patient is necessary for future improvement in care, we must not be distracted and neglect our primary mission to care for all stroke patients including those “not-so-right” – by far, the vast majority for whom we neurointensivists and neurohospitalists currently provide acute stroke care.

## The “Just Right” Recipe: Age, Symptom Severity, and Event Duration

Despite disappointing outcomes, multiple studies within the acute stroke trial literature have nevertheless proven informative. For one, they have identified and highlighted three clinical parameters that repeatedly and reliably impact stroke outcome regardless of intervention: patient age, symptom severity (NIHSS score), and event duration (time). As a result of their clinically predictive value, the stroke community has heavily weighted its determination of endovascular eligibility on these criteria. Strokes of higher severity (NIHSS score >10 and/or consistent with a large-vessel occlusion), shorter duration ( <4.5 h), and in younger patients ( <80 years) have been preferred for trial selection. On the other hand, those of lower severity and longer duration especially in older patients have been considered less preferred for enrollment. At first glance, this inclusion/exclusion “just right” criterion appears reasonable especially in consideration of its reflection of and adherence to our field’s current prevailing theoretical framework of ischemia: that is, ischemic burden is a direct, absolute function of time, and hence, “time is brain.” However, if this set of criteria and model of ischemia that it reflects really do offer a best determination of “just right” endovascular candidacy, then why have our trials not proven this so? The answer certainly cannot entirely lay in device or operator limitation or inadequacy. Completed trials have demonstrated that a variety of endovascular therapies and their operators have consistently yielded revascularization success rates higher than those of t-PA alone particularly for large-vessel occlusions (5, 8, 9). Furthermore, they have achieved these rates without compromising safety (4, 8, 9). Additionally, if these criteria and model do select the patients that really are “just right,” why do these patients, more often than not, require the most intensive and extended hospital stroke care? If they were truly the best candidates for these therapies, should these patients not respond accordingly, and expectedly require less intensive care and shorter hospital stays than their counterparts who were “not-so-right?” Such results provide need to search deeper to understand and explain this puzzling disconnection between excellent revascularization outcomes and poor clinical ones for acute stroke endovascular treatments.

## Time is Brain: A Dead-End in the Goldilocks Dilemma

We consequently direct our attention once again to the “just right” clinical parameters and “time is brain” model of ischemia that provides the basis for their use in determination of endovascular eligibility. In aggregate, age, NIHSS score, and time provide a pre-treatment clinical snapshot that reflects a highly dynamic interplay between baseline health including chronic co-morbidities and acute cerebrovascular pathophysiology. As a real-time estimation of the interaction between acute and chronic illness, these factors provide only that and no more: a generic, over-simplified estimation of active disease. Despite that these factors are clinically generic, we support that they are nevertheless informative and carry utility in the determination of endovascular therapeutic eligibility in acute stroke.

However, this criterion’s application in service of an inadequate model of ischemia has led to an unintentional bias in selection of candidates unlikely to benefit from endovascular treatment and, hence, has resulted in poor trial outcomes. “Time is brain” emphasizes a directly proportional, absolute dependence of mounting ischemia on elapsed time over other contributing factors. In reality, however, the exact milieu of pathophysiology at play in active ischemia is varied, patient-specific and state-dependent: in other words, a highly complex, dynamic process for which an overly simplified model like “time is brain” cannot fully account.

## The “Just Right” Disease State: Acute Stroke Case Illustrations

Let us examine eligibility determination for endovascular treatment in three theoretical cases of acute large-vessel occlusive stroke. The first candidate is a younger patient with a severe stroke of longer duration: within the current framework of eligibility, a poor candidate for endovascular therapy. This patient’s current clinical picture signifies a sizable load of active ischemia sustained over a relatively short period of time. Consequently, it indicates a reduced capacity to tolerate and withstand acute injury of at-risk tissue without symptomatology; and, is reflective of an underlying lack of preexisting disease-mitigating factors, and/or presence of high burden of chronic morbidities, and/or their compounded effects (i.e., peripheral vascular, collateral, and cardiac function statuses). As a result, this patient’s active disease state is not only severe, but also poorly amenable to preserving penumbral tissue and, therefore, salvaging endovascular therapies which target it.

The second theoretical candidate is a younger patient with a severe stroke of shorter duration: within the prevailing “time is brain” paradigm of acute stroke treatment, an excellent candidate who appears “just right” for endovascular therapy. However, in consideration of the related pathophysiology driving his clinical status, we argue that this patient is not only a poor candidate, but, perhaps, even a worse one than the first case. In similar fashion to the first example, this patient’s current clinical state signifies a significant load of active disease. However, more importantly, it has been sustained in an even shorter period of time. This active disease state therefore indicates a profoundly more diminished capacity to tolerate and withstand acute ischemia, and likely is the result of a more pronounced presence of chronic co-morbidities and/or absence of disease-mitigating factors. Consequently, this patient’s active disease state is, in actuality, even more severe; even less favorable to penumbral preservation; and, therefore, even less amenable to salvaging therapies than the first candidate’s.

The final theoretical case is an older patient with a stroke of mild severity and longer duration: by currently applied eligibility guidelines, another relatively poor candidate for endovascular therapy. But, in consideration of the state of active disease that has resulted in this patient’s clinical status, we argue that one should consider him not only a good candidate, but, in fact, as the true “just right” candidate of the three considered. In contrast to the first two examples of large-vessel occlusive stroke, this patient’s current clinical state reflects a minimal load of ischemia but a large area of penumbra preserved despite, strikingly, a longer duration of acute illness. This active disease state consequently indicates an inherently better capacity to tolerate and withstand acute ischemia, and likely is the result of a notable absence of chronic co-morbidities and/or presence of disease-mitigating factors. Consequently, this patient’s active disease status is not only fundamentally different than those of the former cases; but, as a result, also more amenable to endovascular rescue therapies.

## Next Steps: “Not-So-Right” may be “Just Right”

These theoretical case examples that fall along a continuum of age, stroke severity, and duration illustrate our contention that these strong predictors of acute stroke clinical outcome do in fact have potential utility in determining which patients are “just right” for endovascular treatments. However, we propose that our field’s currently favored model of ischemia, “time is brain,” is an inadequate oversimplification of the complex, dynamic pathophysiology in acute stroke. As a consequence, its promotion has led unintentionally to a misinterpretation of these three clinical parameters; subsequent selection bias toward “not-so-right” trial candidates; and ultimately continued stasis in the progress of acute stroke therapy. Therefore, we argue for its replacement with a theoretical framework that better encapsulates and accounts for the dynamic, nuanced complexity of the active ischemic disease state. In support, we provide as a basis for these clinical predictors a framework of ischemia that accounts for the global state of pathophysiology during acute stroke: one that incorporates stroke severity and duration not as independent factors, but as interactive and compounding contributors along a spectrum of active disease states. Indeed, we must be mindful that although severity and duration undoubtedly influence clinical outcomes, so too do a host of other exacerbating and mitigating factors that fall under the umbrella of the active disease state and influence tissue ischemia and viability.

Accordingly, we should give pause to consider whether there may be multiple or, unfortunately, no patients that are “just right” for endovascular therapies. Both possibilities suggest that such a Goldilocks search may be unjustified and not worthwhile. Indeed, the vast majority of stroke patients that require our acute care in the hospital are overwhelmingly “not-so-right” precisely because they represent a broad continuum of active disease states across the myriad of interactive acute and chronic illnesses that can clinically declare themselves as acute cerebrovascular pathology. If we desire to advance the field of acute stroke and its treatments, it is imperative that we not lose sight of this fact. It begs us to remain humble, open-minded, and open-hearted to the notion that our current understanding of acute stroke is still relatively superficial. And with that acceptance comes a hope that, with continued improvement in our understanding, we be able to call fewer and fewer patients “not-so-right” in the future. To this end, we propose that a more nuanced framework of acute ischemia replace our field’s current one to allow for improved interpretation of valid clinical criteria and determination of eligibility for endovascular treatments in acute stroke trials. Such a step gives promise that our Goldilocks search will continue and ultimately, hopefully, identify the ideal acute stroke patient: one whose acute stroke may be small in severity or even long in duration, but whose active disease state, regardless, is just right.

## Conflict of Interest Statement

The authors declare that the research was conducted in the absence of any commercial or financial relationships that could be construed as a potential conflict of interest.
